# An implantable dual-chamber capsule with three release modes for site-specific delivery and enhanced therapeutic efficacy

**DOI:** 10.1016/j.isci.2026.115099

**Published:** 2026-03-06

**Authors:** Limin Ren, Enhe Kou, Wenqiang Zhang, Shixun Li, Xunwei Tong, Liwei Ren, Yisong Tan

**Affiliations:** 1 School of Mechanical Engineering, Northeast Electric Power University, Jilin, Jilin 132012, China; 2 College of Mechanical & Energy Engineering, Beijing University of Technology, Beijing 100124, China; 3 School of Automation Engineering, Northeast Electric Power University, Jilin, Jilin 132012, China

**Keywords:** Drug delivery system, Biotechnology, Biodevices, Materials science, Magnetic materials

## Abstract

Precise and flexible drug delivery is increasingly required in clinical therapy, yet most implantable systems remain constrained by limited selectivity, low loading capacity, and bulky size. Here, we present an implantable capsule that employs magnetically actuated vibration of an internal metallic cantilever to achieve magnetically controlled release. The capsule integrates a dual-reservoir structure, enabling the device to load two drugs and support multimodal release through three independent modes. Experimental evaluation demonstrates a drug retention rate of 76% and a single release volume of 0.01 mL with fluctuations within ±0.0005 mL, while maintaining a miniature form factor (13 × 5 × 5 mm^3^) and stable performance under thermal (45 °C) and mechanical (50 g) stress. *In vivo* studies further confirm that insulin and lidocaine hydrochloride delivered by this capsule achieve therapeutic outcomes comparable to subcutaneous injection. Combining compactness, robustness, and multi-mode controllability, this system supports long-term implantable drug administration.

## Highlights

• Dual-reservoir magnetostrictive capsule enables wireless AMF-triggered drug release
• Cantilever frequency selection enables reliable switching among three release modes
• Miniature 13 × 5 × 5 mm^3^ capsule achieves76% drug loading with repeatable dosing
• Stable release under depletion, 45°C heating and 50 g load

## Graphical abstract

**Figure:**
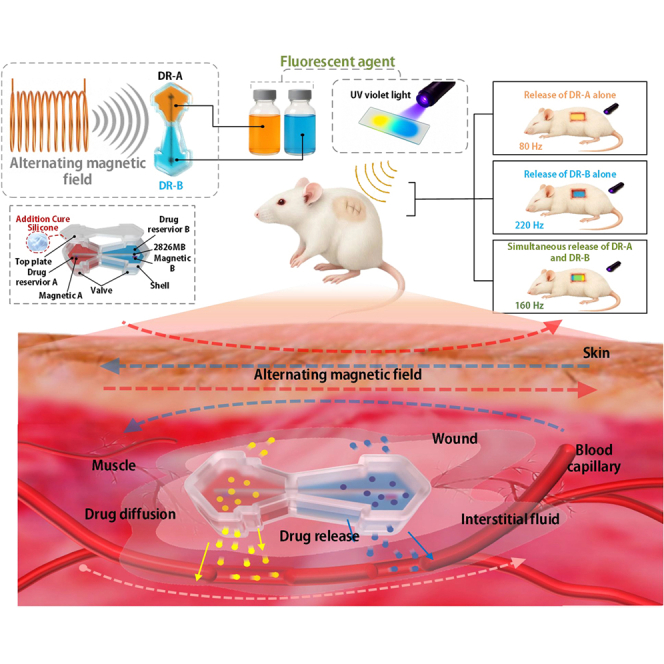

## Introduction

Chronic diseases in elderly populations often present with multiple symptoms, making conventional regimens of frequent oral and injectable administration burdensome.^1^ Implantable drug delivery devices capable of addressing multiple conditions, therefore, offer an effective alternative.^2^

As an implantable device for *in vivo* drug release, sufficient drug storage capacity within a compact footprint is essential. Zheng et al. reported a magnetically driven capsule with two internal magnetic balls (Φ6.3 × 12.3 mm^3^), where the drug is stored only at the tip, resulting in 9.9 μg per release despite a theoretical 180 actuations, which is inadequate for clinical dosing.^3^ Lee et al. developed a miniature implantable pulsatile device that stores 1 mL of 5-FU solution (10 mg/mL) in a total volume of 3.3 mL, corresponding to about one-third of the device volume.^4^ Costello et al. proposed a degradable 6 mm square implant with a dexamethasone loading ratio of 60%, but its degradation-dependent release mechanism limits dose precision.^5^ Picco et al. demonstrated a piezoelectric valve-based system that enables accurate dosing, yet its large, wired configuration restricts it to micro-dosing rather than long-term, fully implantable use.^6^ Zeng et al. designed a flexible, fluid-driven device that releases a drug by filling its middle layer, but it swells to 1.5–2 times its original size during actuation, requiring a larger incision and potentially complicating recovery.^7^ Ye et al. developed a magnetically controlled dual-chamber capsule with a pneumatic reservoir (0.6 mL total, 0.3 mL per chamber), but its 29 mm × 13 mm footprint occupies a relatively large subcutaneous area.^8^ Young Bin Choy et al. introduced a magnetically actuated pump with a 3 mL reservoir that meets long-term insulin therapy requirements; however, its total volume of 7.2 cm^3^ and 30 mm length yield a storage ratio of only 41.2% and may cause discomfort during implantation.^9^ These examples highlight the need for implantable systems that simultaneously maximize relative storage capacity and minimize overall device size.

In disease treatment with implantable drug delivery systems, relying on a single drug often fails to achieve optimal therapeutic outcomes.^10^ Recent magnetically actuated implantable systems have emerged as a promising strategy for wireless control of therapeutic functions *in vivo*.^11^^,^^12^ These studies demonstrate that external magnetic fields can reliably trigger drug release from miniaturized implants in a remotely controlled manner.^13^^,^^14^ Most of these systems remain single-reservoir or single-mode. Lee et al. introduced a self-unrolling sheet in which the drug is stored in a capsule and delivered via a tether, but it permits only four release cycles and is therefore unsuitable for long-term therapy.^15^ Li et al. 3D-printed a dual-drug scaffold whose release depends on *in vivo* cellular degradation; the soft structure also risks leakage under subcutaneous compression.^16^ Kim et al. proposed a multi-unit protein carrier (1.5 g) with a 50% loading ratio, yet its NIR-triggered release requires complete discharge of each unit, limiting dose control.^17^ Koo et al. developed a degradable tri-reservoir device driven by electrochemical valves, but it is assembled from three separate units, offering limited storage and requiring a relatively large implantation wound.^18^ Qi et al. reported a liquid-metal nanoparticle-based platform that enables sequential multi-drug release within a single device, but its storage capacity remains low, and implantation is still invasive.^19^ These limitations highlight the need for implantable systems with versatile, controllable multi-drug release to improve therapeutic performance.

In clinical applications, ensuring user safety is a critical requirement for implantable medical devices.^20^ Implantable drug delivery systems must therefore achieve precise dosing while keeping structural risk low.^21^ Evans et al. reported a piezoelectric valve device equipped with multiple sensors that enables accurate dosing, but it requires substantial electrical power and wired connections to the exterior, reducing user convenience.^22^ Levy et al. developed a battery-powered system driven by button cells, which offers high precision in dose control but carries a risk of severe secondary injury if structural failure occurs *in vivo*.^23^ Hu et al. proposed a magnetically actuated, magazine-shaped capsule capable of quantitative release, yet its planar layout necessitates a relatively large incision, and the rigid plates may cause tissue friction or compression during long-term implantation.^24^ Lacovacci et al.’s battery-powered drug delivery device provides precise single-shot release, but its rigid structure and internal power source could lead to serious internal injury if compromised.^25^ Sun et al. developed a magnetically driven magazine capsule delivering 0.1–0.3 mL per dose; however, its rigid, planar design limits drug capacity, requires a large incision, and poses a risk of long-term tissue damage.^26^ Whyte et al. introduced a soft-bodied device in which a fluid-filled interlayer expels the drug; although the compliant structure enhances biocompatibility and accommodates body motion, the expanded volume after filling may enlarge the incision and complicate recovery.^27^ Overall, implantable drug delivery systems must balance precise control with structural safety and long-term comfort throughout patient use.

To address these challenges, we propose an implantable drug-release capsule. The core mechanism relies on the intrinsic resonance frequencies of magnetostrictive materials with different lengths.^28^ Under an external alternating magnetic field, a microscale metallic cantilever composed of magnetostrictive composites undergoes efficient mechanical vibration when the frequency is matched. This vibration drives the release of drugs from the reservoir through a valve into the subcutaneous environment, thereby achieving precise, on-demand controlled delivery.

The contributions of this study are as follows.

(1) We propose a compact implantable dual-chamber capsule (IDRC) for site-specific release. The capsule achieves a relative drug-loading ratio of 76%, representing a 16% improvement compared with Costello’s design,^5^ and is capable of expelling up to 86% of the loaded drug.
(2) The IDRC integrates two drug reservoirs, enabling simultaneous release of two drugs to enhance therapeutic efficacy. In addition, it supports three release modes, offering greater flexibility for long-term treatment regimens.
(3) The proposed IDRC is biocompatible, structurally simple, and functionally stable, thereby ensuring patient safety and comfort during use.

## IDRC working principle and structural design

**Figure 1:**
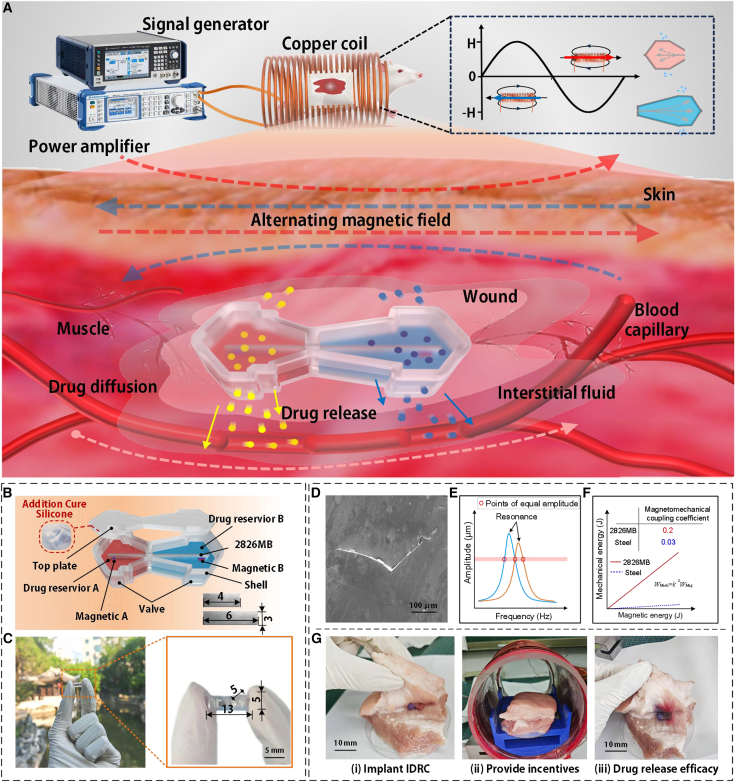
Principle, design, and drug release performance of the IDRC in biological tissue (A) Working principle of the IDRC. (B) Detailed structural design of the IDRC. (C) Photograph of the IDRC prototype. (D) SEM image of the valve. (E) Principle for determining operating frequencies under the three release modes. (F) Comparison of magneto-mechanical coupling coefficients between 2826 MB and steel. (G) Preliminary drug release test of the IDRC implanted in pork tissue.

The proposed IDRC incorporates two internal cantilevers of different lengths as its core components. Figure 1 illustrates the working principle, structural design, and drug release behavior of the IDRC in biological tissue. As shown in Figure 1A, an electrical signal generated by a signal generator is amplified and delivered to a copper coil, producing an alternating magnetic field. When the field frequency matches the natural frequency of a given metallic cantilever, the corresponding cantilever undergoes forced vibration,^29^ with the free end of the cantilever swinging rapidly to eject the drug through the valve and deliver it to the implantation site.

By tuning the output frequency, independent release from each reservoir can be achieved. It should be noted that the IDRC is typically implanted in subcutaneous tissue, which lies beneath the skin and consists of adipocytes,^30^ connective tissue,^31^ blood vessels,^32^ and lymphatics.^33^ Due to the relatively high content of extracellular fluid (interstitial fluid), this environment is moist, and the released drug first diffuses into the interstitial fluid before migrating to the wound site and ultimately entering the vasculature.^34^

As shown in Figure 1B, the IDRC contains two independent dual-chamber reservoirs. Each reservoir is equipped with a 2826 MB metallic cantilever, 4 mm in DR-A and 6 mm in DR-B. The specific parameter information is shown in Table 1. To keep the reservoir volumes equal, DR-A is designed wider than DR-B. At the free end of each cantilever, two small magnets (magnetic A and magnetic B) are attached, providing sufficient amplitude and acceleration during vibration to drive drug release. Valves are installed on both sides of DR-A and DR-B. In the absence of an alternating magnetic field, the drug solution remains confined behind the valve because the elastic restoring force of the valve, together with the slightly sub-atmospheric pressure within the capsule, establishes a sufficiently high opening threshold to prevent passive leakage. When the IDRC is exposed to an alternating magnetic field at its characteristic frequency, vibration of the metallic cantilever induces transient local pressure peaks near the outlet that surpass this threshold, thereby driving the drug through the valve in a well-defined, pulsed manner. The high internal pressure also prevents interstitial fluid from penetrating the valve. The capsule shell is only 0.3 mm thick, allowing the reservoir wall to deform inward as the internal pressure decreases, while achieving a high drug-loading ratio of 76%. In this study, the drug-loading ratio refers to the volume of liquid drug actually loaded relative to the geometric volume of the reservoir cavity.

**Table 1:** Material properties

	DR-A	DR-B
Alloy Grade	Metglas2826MB	
Length	6 mm	4 mm
Width	3 mm	
Thickness	0.001 mm	
Density	7.9 g/cm^3^	
Elastic Modulus	110 GPa	

At the corners, the thickness is increased to 0.4 mm to provide mechanical support and prevent collapse under skin or external pressure. A photograph of the IDRC prototype is shown in Figure 1C, with overall dimensions of 13 × 5 × 5 mm^3^, ensuring minimal physiological impact. The shell is fabricated from addition-cure silicone, offering high softness and patient comfort. The one-way valve is designed as a triangular structure with 500 μm side length, as shown in the SEM image in Figure 1D.

The three drug-release modes of the IDRC are enabled by two metallic cantilever beams of different lengths. Their length mismatch gives rise to distinct natural frequencies and corresponding amplitude-frequency response curves, which intersect at a frequency where the two beams exhibit identical vibration amplitudes; this intersection defines the dual-drug co-release mode, as shown in Figure 1E. Two additional equal-amplitude working frequencies are then chosen on either side of this point, near the resonance peak of the long and short cantilevers, respectively. Thus, the long cantilever dominates at low frequencies and the short cantilever at high frequencies, so that each frequency selectively activates a single drug reservoir and achieves selective drug release.

In selecting cantilever materials, 2826 MB was chosen instead of conventional steel. Figure 1F compares the magnetomechanical coupling coefficient squared of the two materials, showing that 2826 MB provides far more efficient conversion of magnetic energy into mechanical vibration. To further evaluate performance, a preliminary release test was conducted by implanting the IDRC beneath pork tissue. The experimental setup, coil actuation process, and drug release outcome are displayed in Figure 1G, where clear traces of release are visible from both reservoirs under electromagnetic excitation. These results confirm the soundness of the IDRC design and establish a firm basis for subsequent studies and applications.

## Experimental results and discussion

To comprehensively evaluate the drug release performance, long-term therapeutic efficacy, and patient-related safety and comfort of the proposed IDRC, extensive experimental validation was conducted from both *in vitro* and *in vivo* perspectives.

### *In vitro* drug release experiments of the IDRC

**Figure 2:**
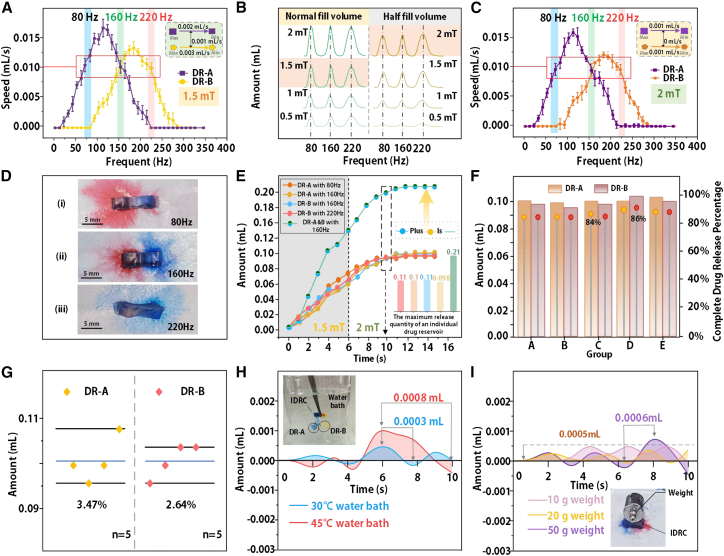
*In vitro* drug release experiments of the IDRC (A) Frequency sweep tests for the two reservoirs of the IDRC. Data are mean ± SD, *n* = 3 technical replicates. (B) Comparison of drug release under normal fill and half fill conditions. Data are mean ± SD, *n* = 3 technical replicates. (C) Frequency sweep of the IDRC at half fill with 2 mT field strength. Data are mean ± SD, *n* = 3 technical replicates. (D) Test of the selective drug release capability of the IDRC. Data are mean ± SD, *n* = 3 technical replicates. (E) Drug release profiles over time under three operating modes. Data are mean ± SD, *n* = 3 technical replicates. (F) Schematic of complete drug release from the IDRC. Data are mean ± SD, *n* = 3 technical replicates. (G) Coefficient of variation for complete drug release at full loading. *n* = 3 technical replicates were performed for 5 IDRC devices. (H) Comparison of release performance at 30°C and 45 °C relative to room temperature. Data are mean ± SD, *n* = 3 technical replicates. (I) Comparison of release performance under applied loads of 10 g, 20 g, and 50 g versus no load. Data are mean ± SD, *n* = 3 technical replicates.

*In vitro* drug release experiments were first conducted to preliminarily test and quantify the release capability of the IDRC. To investigate the release conditions, frequency sweeps from 0 to 350 Hz were applied to the metallic cantilevers in DR-A and DR-B. At each frequency, five repeated trials were performed. The drug release profiles of the two reservoirs at different frequencies are shown in Figure 2A. Maximum release rates were observed at 120 Hz and 190 Hz for DR-A and DR-B, respectively, which coincide with the resonance frequencies of the cantilevers. The two curves intersected at 160 Hz, where the release rate reached 0.01 mL/s. The same release rate was also observed when DR-A operated at 80 Hz and DR-B at 220 Hz. Across five trials, the maximum deviation in release rate was 0.002 mL/s for DR-A and 0.003 mL/s for DR-B, with an average difference of only 0.001 mL/s between the two reservoirs.

During drug release, the release rate decreases as the reservoir volume diminishes. To address this, when the drug volume was reduced to half of the normal level, the magnetic field strength was increased to enhance cantilever vibration and maintain release efficiency. As shown in Figure 2B, the release volume under normal fill with a 1.5 mT field was equivalent to that under half fill with a 2 mT field. The release performance of the IDRC at half fill is presented in Figure 2C. Under these conditions, five repeated trials confirmed that the release rates of DR-A and DR-B matched those observed in Figure 2A, with a maximum deviation of 0.001 mL/s and identical mean values. Based on these results, the IDRC operating modes were defined as follows: selective release from DR-A at 80 Hz, selective release from DR-B at 220 Hz, and simultaneous release from both reservoirs at 160 Hz.

To validate the selective release capability of the IDRC, drug release experiments were performed on moist filter paper, as shown in Figure 2D. In Figure 2D-(i), at 80 Hz, only DR-A released the red agent, while Figure 2D-(iii) shows that at 220 Hz, only DR-B released the blue agent. These results demonstrate that each reservoir responds exclusively to its designated frequency, with no cross-interference. At 160 Hz, as illustrated in Figure 2D-(ii), both DR-A and DR-B are released simultaneously. The distinct color patterns on the filter paper clearly confirm the IDRC’s ability to achieve selective drug release.

Figure 2E presents the drug release profiles over time under the three operating modes. With the reservoirs fully loaded, the IDRC was placed in a 1.5 mT magnetic field, where the cumulative release increased gradually with time. At 6 s, when the reservoir volume was reduced to half, the field was raised to 2 mT. By 10 s, as the reservoirs approached depletion, the release curves leveled off. The results show that the release rate of each reservoir stabilized at approximately 0.01 mL/s, with a maximum difference of 0.012 mL between DR-A and DR-B. In addition, the sum of the values at each node on the DR-A and DR-B curves corresponds to the drug release observed at 160 Hz. The IDRC not only provides a relatively large drug storage capacity but also achieves a high level of drug release. As shown in Figure 2F, five trials were conducted in which all drug contents were released, yielding maximum complete release percentages of 84% for DR-A and 86% for DR-B. The results indicate that the overall release performance was highly consistent across trials. Figure 2G further presents the coefficients of variation calculated from the total expelled volume. The average maximum release was 0.103 mL, with coefficients of variation of 3.47% for DR-A and 2.64% for DR-B. These findings demonstrate that the structural design of the IDRC has minimal influence on the completeness of drug release.

In certain pathological conditions, body temperature may rise to extreme levels of up to 43 °C. To verify the stability of the IDRC under such conditions, a 10 s release test was conducted simulating elevated physiological temperatures. The experiments were performed in water baths at 30°C and 45 °C, and the results were compared with those obtained under normal conditions at 25 °C on moist filter paper. The differences are plotted in Figure 2H. The data show that fluctuations were 0.0003 mL at 30 °C and increased to 0.0008 mL at 45 °C. This indicates that extreme hyperthermia can cause minor disturbances in the release process, though the overall effect remains negligible. The observed fluctuations are attributed to the 0.3 mm wall thickness of the capsule, which makes it sensitive to temperature changes, and to the critical transition point at 6 s when the magnetic field was reinforced, where a transient peak appeared before stabilizing within 2 s.

Finally, the drug release performance of the IDRC was evaluated under different loading conditions. Weights of 10 g, 20 g, and 50 g were placed on top of the device, and the release amounts were compared with those obtained under normal conditions. In the mechanical stability tests, 10 g and 20 g were used to mimic typical loads from daily posture and mild compression on a subcutaneous implant, whereas 50 g served as an upper-bound load to simulate occasional stronger external compression. The statistical results are shown in Figure 2I. At 10 g and 20 g, only minor fluctuations were observed relative to the no-load case, while a transient peak of 0.0006 mL appeared under 50 g. Unlike the thermal test in Figure 2H, no sharp spike was detected at 6 s. These findings demonstrate that the structural characteristics of the IDRC effectively buffer external loading effects, maintaining drug release within a relatively stable range and ensuring good adaptability to different mechanical stresses.

### *In vivo* fluorescent drug release experiments of the IDRC

**Figure 3:**
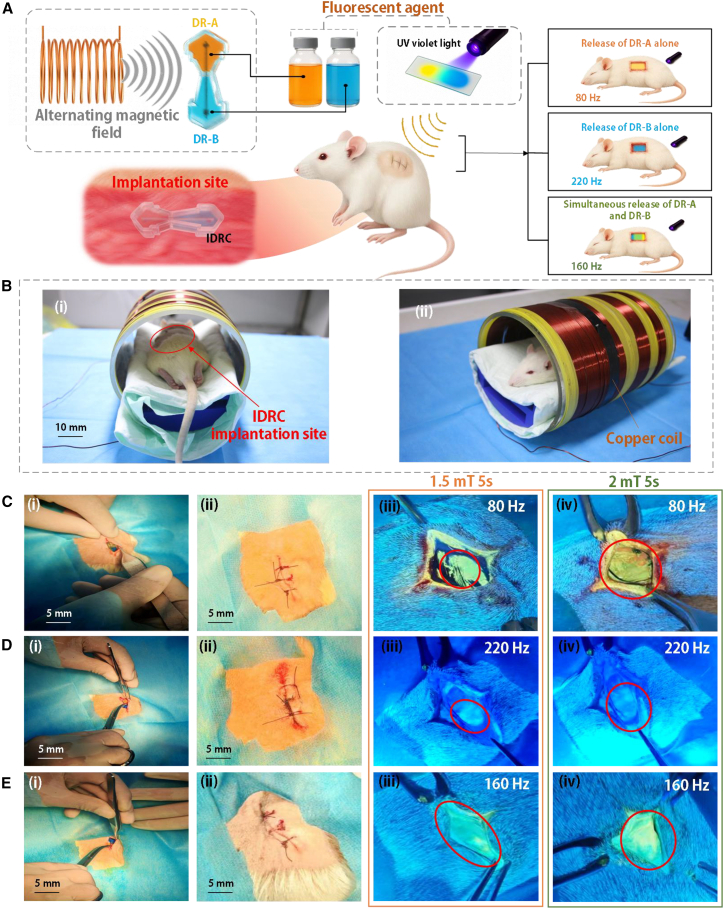
*In vivo* fluorescent drug release experiments of the IDRC (A) Working principle of the *in vivo* release experiment. (B) Implantation procedure of the IDRC in rats. (C) Selective release from DR-A at 80 Hz. Data are mean *n* = 1 technical replicate were performed for 3 rats. (D) Selective release from DR-B at 220 Hz. Data are mean *n* = 1 technical replicate were performed for 3 rats. (E) Simultaneous release from DR-A and DR-B at 160 Hz. Data are mean *n* = 1 technical replicate were performed for 3 rats.

To investigate the *in vivo* release behavior of the IDRC, the device was implanted subcutaneously in adult rats. Fluorescent agents were used to visualize drug release under different modes, with distinct colors corresponding to each release pathway. This approach further validated the independent release capability of the three modes. The experimental procedure and representative results are shown in Figure 3.

Figure 3A illustrates the working principle of the *in vivo* drug release experiment. DR-A and DR-B were loaded with yellow and blue fluorescent agents, respectively, and the IDRC was implanted subcutaneously in rats before being placed inside an electromagnetic coil to trigger release. At 80 Hz and 220 Hz, the IDRC released the yellow and blue agents individually, while at 160 Hz, both were released simultaneously, producing a green signal where the two colors overlapped. The emitted fluorescence was clearly visible under UV violet light. Figure 3B shows the implantation procedure, with Figure 3B-(i) indicating the actual subcutaneous position of the IDRC and Figure 3B-(ii) depicting the rat positioned inside the copper coil.

Figure 3C shows the drug release process of the IDRC under 80 Hz excitation. In Figures 3C-(i) and 3C-(ii), the IDRC loaded with two fluorescent agents was implanted subcutaneously in rats and sutured. Under the alternating magnetic field, the yellow fluorescent agent from DR-A was released. Figure 3C-(iii) demonstrates that after the first release, the yellow fluorescence covered nearly two-thirds of the wound, while Figure 3C-(iv) shows that after the second release, the fluorescence had fully spread across the wound. Figure 3D presents the process at 220 Hz. Under otherwise identical conditions, the blue fluorescent agent from DR-B was released. In Figure 3D-(iii), the blue fluorescence already covered more than half of the wound after the first release, and Figure 3D-(iv) illustrates that after the second release, the wound was completely filled. Figure 3E illustrates the results at 160 Hz. After the first release, Figure 3E-(iii) shows that the green fluorescence, generated by the combination of yellow and blue agents, covered almost the entire wound, and Figure 3E-(iv) confirms that after the second release, the wound was completely filled with green fluorescence.

Figure 3C shows the drug release process of the IDRC under 80 Hz excitation. In Figures 3C-(i) and 3C-(ii), the IDRC loaded with two fluorescent agents was implanted subcutaneously in rats and sutured. Under the alternating magnetic field, the yellow fluorescent agent from DR-A was released. Figures 3C-(iii) demonstrates that after the first release, the yellow fluorescence covered nearly two-thirds of the wound, while Figure 3C-(iv) shows that after the second release, the fluorescence had fully spread across the wound. Figure 3D presents the process at 220 Hz. Under otherwise identical conditions, the blue fluorescent agent from DR-B was released. In Figure 3D-(iii), the blue fluorescence already covered more than half of the wound after the first release, and Figure 3D-(iv) illustrates that after the second release, the wound was completely filled. Figure 3E illustrates the results at 160 Hz. After the first release, Figure 3E-(iii) shows that the green fluorescence, generated by the combination of yellow and blue agents, covered almost the entire wound, and Figure 3E-(iv) confirms that after the second release, the wound was completely filled with green fluorescence.

The *in vivo* fluorescent drug release experiments demonstrate that the IDRC, as an implantable device, enables controlled and precise drug delivery. In addition, the cumulative release amount increased significantly with successive release events.

### Pharmacological efficacy of the IDRC versus conventional drug administration

**Figure 4:**
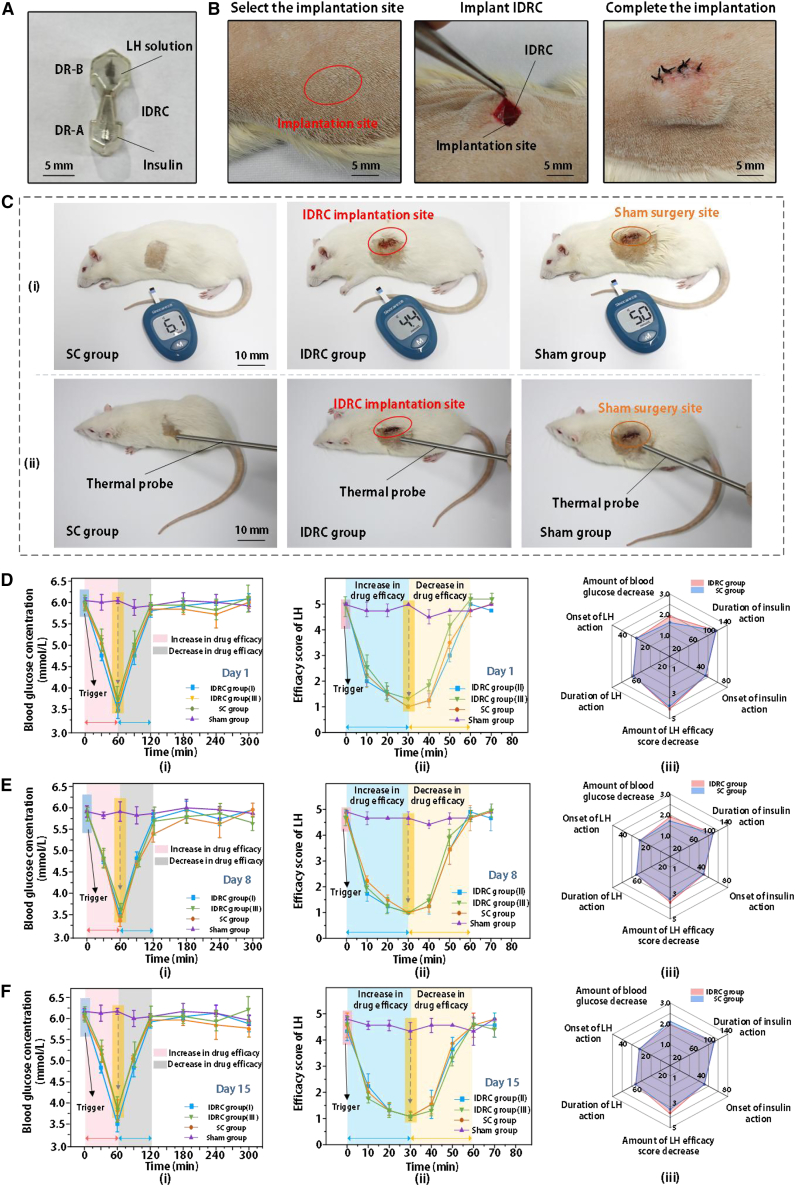
Comparison of pharmacological efficacy between the IDRC and conventional administration (A) Photograph of the IDRC loaded with insulin and lidocaine hydrochloride. (B) Implantation procedure of the drug-loaded IDRC in rats. (C) Blood glucose and sensitivity assessment in three groups of rats. (D–F) Pharmacodynamic performance of the two drugs on days 1, 8, and 15. Data are mean ± SD. *n* = 1 technical replicates were performed for 3 rats.

The previous experiments confirmed that the proposed IDRC is capable of selective drug release. In this section, insulin and lidocaine hydrochloride (LH) solutions were loaded into the IDRC and implanted subcutaneously in healthy adult rats. By comparing the pharmacodynamic equivalence of insulin and LH delivered through the IDRC with that of conventional subcutaneous injection, we evaluated the feasibility of the IDRC as an alternative to traditional administration methods. The experimental results are shown in Figure 4. The therapeutic effects of insulin and LH were assessed by monitoring blood glucose levels and by scoring locomotor performance after LH administration,^35^ with the evaluation criteria summarized in Table 2.

**Table 2:** Locomotor performance scoring of rats after lidocaine hydrochloride administration

Score	Response description
1	No observable response
2	Lifts foot or exhibits slight twitching
3	Withdraws foot and turns head toward the stimulus site
4	Lifts paw and licks the site
5	Rapid escape

As shown in Figure 4A, DR-A was loaded with 0.35 mg/mL insulin and DR-B with 2 mg/mL LH solution. The drug-loaded IDRC was then implanted subcutaneously in rats, as illustrated in Figure 4B. Figure 4C presents the evaluation of blood glucose and sensitivity in three groups of rats: the implantation group, the control group, and the sham group.^36^ In the control group, rats received equivalent doses of drugs via subcutaneous (SC) injection, while in the sham group, a surgical incision was made without drug administration. Before each experiment, rats were placed in a magnetic field for 1 s to trigger drug release from the IDRC, while SC group rats were injected with 0.01 mL of drug, and sham group rats underwent incision only. Blood glucose levels and sensitivity were subsequently measured in each group. Figure 4C-(i) shows the experimental procedure for evaluating insulin efficacy using a glucometer, while Figure 4C-(ii) depicts the test of LH efficacy by applying thermal stimulation at 50 °C.

The pharmacodynamic performance of insulin and LH in rats on days 1, 8, and 15 is shown in Figures 4D–4F. On day 1 (Figure 4D), insulin was released for 1 s at 80 Hz, and blood glucose was measured every 30 min. As illustrated in Figure 4D-(i), the IDRC and SC groups exhibited similar trends, with blood glucose reaching a minimum of 3.4–3.8 mmol/L at 60 min and returning to baseline by 120 min, while the sham group remained around 6 mmol/L. Figure 4D-(ii) shows the sensitivity test results when LH was released for 1 s at 220 Hz and measured every 10 min. Both IDRC and SC groups followed comparable patterns, reaching the lowest efficacy score of 1 at 30 min, indicating maximum anesthetic effect, which gradually diminished and disappeared by 60 min. Figure 4D-(iii) compares the effects of insulin and LH delivered by IDRC and SC injection, revealing similar pharmacodynamic profiles. Results from day 8 and day 15 are presented in Figures 4E and 4F, respectively. Compared with day 1, the durations of efficacy onset and decline remained stable, and the peak effects of both drugs were consistent, demonstrating reliable stability over time. Figure 4D–4F-(iii) further shows that the pharmacological performance of IDRC closely matched that of SC injection, confirming the feasibility of IDRC as an alternative to conventional administration.

These experiments further confirm the precision and reliability of the IDRC in drug release, highlighting its potential as an alternative to conventional delivery methods and providing experimental evidence for future clinical applications.

### Biocompatibility and biosafety assessment of the IDRC

**Figure 5:**
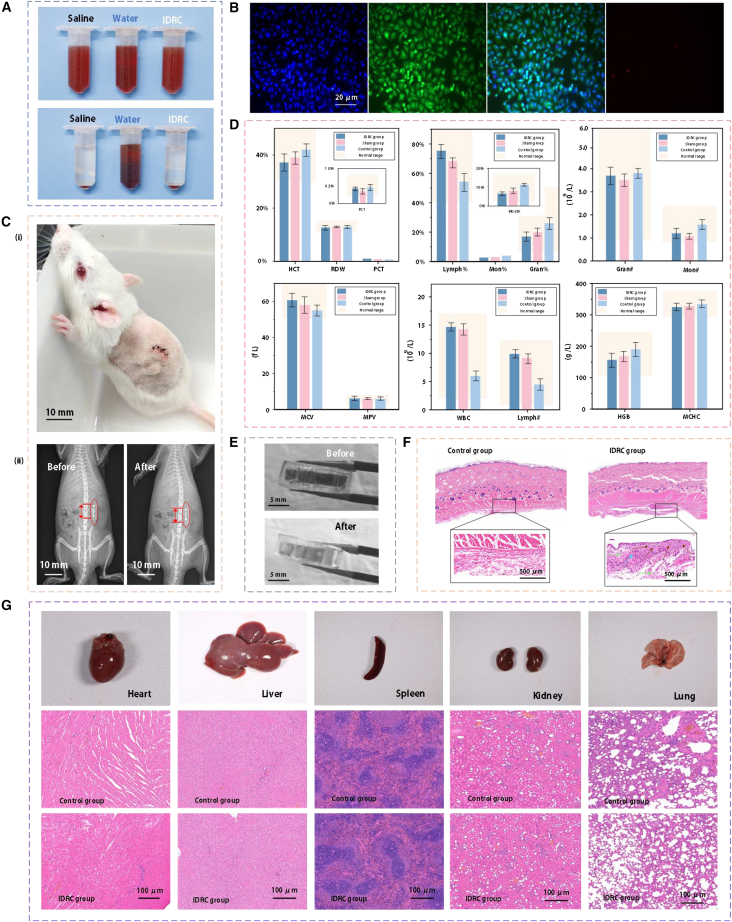
Biosafety evaluation of the IDRC and corresponding results (A) *In vitro* hemolysis test of the IDRC material. (B) Fluorescent staining of cells cultured on the IDRC material substrate. (C) Physiological condition of rats and X-ray imaging during the implantation period. (D) Complete blood count analysis of experimental rats. Data are mean ± SD. *n* = 6 technical replicates were performed. (E) Comparison between unused IDRC and IDRC after implantation. (F) Histological sections of implantation sites compared with control tissue. (G) Histological sections of major organs from implanted rats.

To ensure the long-term applicability of the IDRC as an implantable device, its biosafety was evaluated through histopathology, blood analysis, and immune response, with the experimental procedures and results summarized in Figure 5. In Figure 5A, the primary material of the IDRC (addition-cure silicone) was subjected to a hemolysis test.^37^ After centrifugation, the supernatant remained clear and transparent, with red blood cells deposited at the bottom of the tube, resembling the negative control. This indicates that the material does not cause significant red blood cell rupture and exhibits negligible hemolytic activity, confirming good blood compatibility. In addition, live/dead cell fluorescent staining was performed on cells cultured on the material substrate, as shown in Figure 5B.^33^ The results revealed high cell viability (strong Calcein AM signals widely distributed) and very low cell mortality (few PI-positive signals). DAPI staining showed regular nuclear morphology with uniform distribution, suggesting healthy cell growth. Calcein AM staining further indicated strong cytoplasmic metabolic activity. Together, these results demonstrate that the material used in the IDRC does not release cytotoxic substances that would inhibit cell growth.

Figure 5C shows the physiological condition of rats and X-ray results during the implantation period.^38^ Figure 5C-(i) presents the condition of a rat on day 15 after subcutaneous implantation of the IDRC. Throughout the 15-day period, apart from mild lethargy in the initial days following implantation, the rats exhibited no significant changes in overall condition compared with the pre-implantation state. During this period, no abnormalities in locomotion or feeding behavior were observed. X-ray images taken at the beginning and end of the observation period, as shown in Figure 5C-(ii), further confirmed that the IDRC maintained a stable position *in vivo* without noticeable displacement.

After the completion of the implantation period, whole blood samples were collected from the experimental rats for analysis. The results, shown in Figure 5D, were compared with those from the sham and control groups. White blood cell counts (WBC = 14.8 × 10^9^/L) were at the upper end of the reference range (1.9–16.8 × 10^9^/L), indicating mild immune activation. The absolute lymphocyte count (Lymph# = 10 × 10^9^/L) was also near the upper limit (0.91–12.2 × 10^9^/L), with lymphocyte percentages remaining elevated, reflecting a lymphocyte-dominant immune response. In contrast, neutrophils (Gran# = 3.5 × 10^9^/L) and monocytes (Mon# = 0.6 × 10^9^/L) were within normal ranges, showing no hematological evidence of acute or chronic inflammation. Red blood cell indices were normal: hemoglobin (HGB = 150 g/L) was mid-to-high within the reference interval, and hematocrit (HCT), mean corpuscular volume (MCV), mean corpuscular hemoglobin (MCH), mean corpuscular hemoglobin concentration (MCHC), and red cell distribution width (RDW) all fell within normal values, confirming stable hematopoiesis and oxygen transport. Platelet counts (PLT = 780 × 10^9^/L) and related indices (MPV, PDW, PCT) were also normal, with no signs of platelet activation or coagulation abnormalities. Collectively, these findings indicate that IDRC implantation for 15 days induced only a mild, lymphocyte-driven immune response without neutrophil or monocyte elevation, and both erythrocyte and platelet systems remained unaffected. This confirms good blood compatibility of the IDRC, with a minimal and controllable immune reaction consistent with expected implant-associated responses. At the end of the observation period, the implanted IDRC was retrieved and compared with an unused device, as shown in Figure 5E.^39^ No differences in appearance or integrity were observed, demonstrating that the IDRC remained stable and unaffected by the host environment.

To evaluate the local tissue response induced by the IDRC, samples from the implantation site were collected to assess inflammation and fibrosis. As shown in Figure 5F, compared with the control group, mild fibrous tissue proliferation of approximately 250 μm (orange arrows) was observed, accompanied by a small number of lymphocytes (green arrows) and granulocytes (cyan arrows).^40^ These findings indicate that the IDRC did not elicit severe acute inflammation or marked tissue rejection, demonstrating good biocompatibility.

Subsequently, H&E staining was performed on major organs from the experimental and control groups to assess whether IDRC implantation induced systemic toxicity or pathological damage, as shown in Figure 5G.^41^ The histological results revealed no significant differences in the morphology of key organs (heart, liver, spleen, kidney, and lung) between implanted and control rats. Both groups maintained normal cellular and tissue architecture, with no evidence of necrosis, inflammatory infiltration, or structural disruption. These findings demonstrate that IDRC implantation did not cause detectable pathological alterations in major organs. Overall, the IDRC exhibited good biocompatibility and safety at the cellular, local tissue, and systemic levels.

In summary, these experiments comprehensively verified that the proposed IDRC enables precise drug release, ensures safety, and supports long-term administration, thereby providing a critical biosafety foundation for its long-term implantable application.

### Comparison with similar studies

The IDRC was compared with representative implantable drug-delivery systems across actuation mechanisms, structural design, drug-loading capacity, and release behaviors. The comparative results are presented in Tables 3 and 4. The comparison shows that the IDRC, driven by an alternating magnetic field, achieves stable multi-mode release within a compact dual-reservoir structure. Its simple yet precise actuation and reliable controllability outperform existing magnetic and electromechanical systems, while its multi-mode design concept offers insight for future development of adaptive drug-delivery devices.

**Table 3:** Comparison of actuation mechanisms and structural features

Project	Type	Stimulation Method	Magnetic Field Intensity	Main Structure	Size	Multi-drug
IDRC	Implantable	Alternating magnetic field	2 mT	Dual-chamber cantilever beam encapsulated in elastomer	13 × 5 × 5 mm^3^**⬆**	Dual-drug/three-mode release**⬆**
[Zheng et al.^3^]	Implantable	Permanent-magnet-triggered bistable ball valve	90–140 mT	Dual magnetic balls + check valve	Φ 6.3 × 12.3 mm	Single drug
[Costello et al.^5^]	Implantable	Degradation	–	Polymer rod body	Ø 6 × 0.46 mm	Single drug
[Zeng et al.^7^]	Implantable	Electromagnetic hybrid stimulation	∼30 mT	Microarray liquid-storag0e chambers	–	Multi-drug/sequential
[Ye et al.^8^]	Ingestible	Static magnetic field-driven rotation	0.9–1.2 T	Dual chambers + airbag puncture	Φ 13 × 29 mm	Dual drug
[Iacovacci et al.^25^]	Implantable	Motor + magnetic docking control	100 mT	Micropump system + refill port	78 × 63 × 35 mm	Single drug
[Sun et al.^26^]	Implantable	Alternating magnetic field	30 mT	Soft magnetic valve leaf + elastic encapsulation	Capsule-scale	Single drug

**Table 4:** Comparison of release performance and *in vivo* evaluation

Project	Single Dose	Dose Precision	Number of Releases	Animal Model & Duration	*In vivo* Evaluation
IDRC	0.01 mL ± 0.0005 mL**⬆**	CV ≈ 3%**⬆**	30 times (three-mode switching)	Subcutaneous implantation in SD rats for 15 days	Insulin and lidocaine efficacy ≈ subcutaneous injection
[Zheng et al.^3^]	9.9 ± 0.17 μg per dose	CV ≈ 3.5%	180 times (stable cycles)	Subcutaneous implantation in SD rats for 15 days	PK/PD comparable to subcutaneous injection
[Costello et al.^5^]	0.7 mg solid-loaded	Uncontrolled	One continuous release (passive)	Subcutaneous implantation in SD rats for 30 days	Sustained-release control group
[Zeng et al.^7^]	50–100 μL per dose	Programmed control	10 times (sequential multi-reservoir)	Subcutaneous implantation in rats for 21 days	Multiple programmed administrations successful
[Ye et al.^8^]	0.3 mL per dose	±5%	2 times (dual-target release)	Acute gastric cavity experiment in pigs	Verified synchronized dual-target release
[Iacovacci et al.^25^]	3.18 μL ± 0.30 μL	–	50 times	Abdominal implantation in pigs for 60 days	Stable control of insulin concentration
[Sun et al.^26^]	≈10 μL per dose	CV ≈ 4%	10 times	Simulated *in vivo* pig gastric cavity	Real-time endoscopic response verified

This study introduces an implantable dual-chamber capsule with three distinct release modes for site-specific delivery and improved therapeutic efficacy. The device achieves a high drug-loading ratio of 76% and enables controlled release through magnetically actuated cantilever vibration. It can accurately release 0.01 mL per actuation and maintains stable performance under thermal (45 °C) and mechanical (50 g) stress with variation within ±0.0005 mL. *In vivo* experiments demonstrated that insulin and lidocaine hydrochloride delivered by the IDRC achieved pharmacodynamic effects comparable to subcutaneous injection, independent of release mode. Together, these results highlight the IDRC as a safe, precise, and adaptable platform, offering strong potential as a long-term alternative for drug infusion and an enabling technology for precision medicine. With rational drug combinations and optimized release strategies, the IDRC shows significant potential in the field of precision medicine.

### Magnetic field safety and regulatory compliance

The actuation field amplitude used in this study (1.5–2 mT at tens to hundreds of hertz) lies well below the exposure limits for low-frequency magnetic fields recommended by ICNIRP and IEEE, supporting the safety and clinical feasibility of the proposed external driver.^42^

### Limitations of the study

Notably, the IDRC offers a high relative drug loading capacity and demonstrates favorable drug release performance. However, as an implantable device, its size may not satisfy the requirements of certain future application scenarios. Future work will focus on device miniaturization and structural optimization, and will further provide a quantitative analysis of the release differences arising from the physical properties of different drugs.

## Resource availability

### Lead contact

Yisong Tan tanyisong@neepu.edu.cn.

### Materials availability

This study did not generate new unique reagents.

### Data and code availability

• All data reported in this article will be shared by the lead contact upon request.
• This study did not generate new code.
• Any additional information required to reanalyze the data reported in this article is available from the lead contact upon request.

## Acknowledgments

This work was supported by the 10.13039/501100001809National Natural Science Foundation of China (No. 52205302), the 10.13039/100007847Natural Science Foundation of Jilin Province (20230101321JC), and the Science and Technology Program of the 10.13039/501100010211Education Department of Jilin Province (JJKH20230108KJ, JJKH20230109KJ). The asterisk indicates the author to whom all correspondence should be directed.

## Author contributions

L. R.: conceptualization. E. K.: investigation, experiment, and writing - original draft. W. Z.: investigation. S. L.: investigation. X. T.: software. L. R.: investigation. Y. T.: supervision and writing - review and editing.

## Declaration of interests

The authors state that they have no financial conflicts of interest or personal relationships that could potentially impact the research presented in this article.

## STAR★Methods

### Key resources table

**Table:** 

REAGENT or RESOURCE	SOURCE	IDENTIFIER
**Chemicals, peptides, and recombinant proteins**		

fluorescent agents	Wanglebao, WLB-100, Shandong, China	N/A
hematoxylin and eosin	Servicebio Technology Co., Ltd	Cat# G1076; RRID: N/A
Calcein-AM	Servicebio Technology Co., Ltd	Cat# G1707-100T; RRID: N/A
Propidium Iodide	Servicebio Technology Co., Ltd	Cat# G1021; RRID: N/A
Insulin	Servicebio Technology Co., Ltd	item code VL-7516; RRID: SCR_003036
lidocaine hydrochloride	Servicebio Technology Co., Ltd	Veterinary Drug Product Approval No. 041535235
penicillin–streptomycin	Servicebio Technology Co., Ltd	Cat# G4015; RRID: N/A

**Experimental models: Cell lines**		

NIH/3T3 fibroblasts	OriCell	Cat# M5-0301; RRID: CVCL_0594

**Experimental models: Organisms/strains**		

Rat: Sprague–Dawley (SD)	Servicebio Technology Co., Ltd	Cat#: GA1204; RRID:RGD_10046

### Experimental model and study participant details

#### Animals

All animal procedures were performed under the supervision and approval of the Animal Experimentation and Use Committee of Northeast Electric Power University (Approval No.: NEEPU-IACUC-2024-0106) in a licensed animal facility (Laboratory Animal License No.: SYXK (Hubei) 2023-0101). Sprague–Dawley (SD) rats (male, 8 weeks old, 300 ± 30 g; Servicebio Technology Co., Ltd., China) were used for all *in vivo* experiments, and no genetically modified animals were involved. Rats were housed under controlled temperature and humidity conditions with free access to food and water.

#### Sex as a biological variable

Only male animals were used in this study. Because device actuation and dosing are determined by the mechanical design, animal sex is not expected to materially affect the device’s normal operation in the context of the functionality tests performed here. Nonetheless, potential sex-associated differences in downstream biological responses were not assessed.

#### Other experimental models/study participants

In the cytotoxicity assay, murine NIH/3T3 fibroblasts (ATCC, CRL-1658; RRID: CVCL 0594) were used as the *in vitro* cell model. Cells were cultured in high-glucose Dulbecco’s modified Eagle’s medium (DMEM; Servicebio, G4550) supplemented with 10% fetal bovine serum (Servicebio, G8003) and 1% penicillin–streptomycin (Servicebio, G4015), and incubated at 37 °C in a humidified atmosphere with 5% CO_2_.

### Method details

#### *In vitro* drug release performance of the IDRC

The drug-loaded IDRC was placed in an alternating magnetic field with frequencies increased from 0 to 350 Hz to determine the operating frequencies for different release modes. Before and after each release operation, the mass of the IDRC was measured using an electronic balance (SUNNE, SN-FA2004, Shanghai, China) to calculate the amount of drug released. For experiments evaluating the effect of temperature, water at the designated temperature was poured into an inverted beaker, and the IDRC was placed in the beaker with tweezers for testing.

#### *In vivo* drug release experiments of the IDRC

The IDRC loaded with yellow and blue fluorescent agents (Wanglebao, WLB-100, Shandong, China) was implanted subcutaneously in rats. A 20 mm incision was made during implantation to allow direct observation. The experiment was divided into three groups corresponding to the IDRC’s three release modes: 80 Hz, 220 Hz, and 160 Hz (single release from each reservoir and simultaneous release from both reservoirs). During the first release, the magnetic field strength was set to 1.5 mT and applied for 5 s. Thirty minutes after the release, the dorsal incision was reopened and the diffusion of fluorescent agents was observed under UV light. After the first observation, the incision was surgically sutured, and a second release was performed 2 h later. Since the reservoirs contained half of the initial volume after the first release, the field strength was increased to 2 mT for the second release, with the same 5 s duration. Thirty minutes after the second release, the incision was reopened again, and the distribution of fluorescent agents was examined under UV illumination to compare the diffusion patterns between the two releases. Fluorescent coverage images were recorded using a digital camera (Nikon D7000, Japan).

#### Pharmacological efficacy of the IDRC versus conventional drug administration

*In vivo* pharmacokinetics and pharmacodynamics of the IDRC were assessed by monitoring blood glucose response to insulin and the anesthetic effect of lidocaine hydrochloride. In the first experiment, rats were divided into four groups: SC insulin injection (SC group), IDRC with insulin release (IDRC group I), IDRC with simultaneous release of insulin (0.35 mg/mL) and lidocaine (IDRC group III), and a sham group. After implantation and 1 s actuation, blood glucose was measured every 30 min for 300 min; SC and sham groups received insulin or saline injections, respectively. In the second experiment, rats were divided into SC lidocaine injection (SC group), IDRC with lidocaine release (IDRC group II), IDRC with simultaneous release of insulin and lidocaine (IDRC group III), and sham groups. After 1 s actuation, rats underwent 50 °C thermal stimulation every 10 min, and locomotor responses were scored.

#### Biocompatibility and biosafety assessment of the IDRC

Cytotoxicity study of IDRC was performed using NIH/3T3 fibroblasts. The cells incubated with leach liquor of IDRC were stained using a Calcein-AM/PI kit. The images of stained cells were captured by the fluorescence microscope (Nikon ECLIPSE Ti, Japan). After 14 days of implantation, the IDRC and sham groups were humanely euthanized. At the terminal time point, orbital venous blood was collected for whole blood analysis. Tissue samples in contact with the IDRC, along with major organs, were harvested and fixed using a tissue fixative (Servicebio, G1076, Hubei, China). Samples were then embedded with an embedding machine (JunJie Electronics, JB-P5, Wuhan, China). Sections were cut into 5 μm slices using a microtome (Leica Instruments, RM2016, Shanghai, China). Following paraffin section dewaxing and cryosection rewarming fixation, hematoxylin and eosin (H&E) staining (Servicebio, G1076, Hubei, China) was performed. The stained samples were examined using an upright microscope (Nikon, Eclipse E100, Japan).

#### Materials

The main body of the IDRC was fabricated from addition-cure silicone (Positon, Guangdong, China) using a molding process. The metal cantilever inside the core mechanism is made of 2826 MB metal material, and its profile is processed by EDM. The magnets used are supplied by Holi Magnet Industry Co., Ltd. (Dongguan, Guangdong, China). After external assembly of the metal cantilever and the magnets at both ends, the entire component is inserted into the main housing of the IDRC, and a layer of liquid gel is applied over the joints to reinforce the connection and improve sealing. The dimensions of the metal cantilever are shown in Table 1. The valve orifice was prepared using a standardized puncturing process. Briefly, a metal needle with a diameter of approximately 100 μm and the silicone membrane were mounted on the upper and lower grips of a materials testing machine (Instron 5543A, Boston, MA, USA). The needle was advanced into the IDRC shell at a speed of 0.1 mm/s to a depth of about 3 mm and then withdrawn. This procedure was repeated at the three designated vertex positions of the triangular valve orifice, thereby forming a precise triangular valve structure.

### Quantification and statistical analysis

Statistical analyses were performed using Origin (OriginLab, USA). Quantitative data are presented as mean ± standard deviation (SD), with the mean as the measure of central tendency and SD as the measure of dispersion. Comparisons between two groups were conducted using a two-tailed Student’s *t* test.

The exact value of n and what n represents are provided in the corresponding figure legends.

#### Ethics statement

All animal experiments in this study were conducted under the supervision and with the approval of the Animal Experimentation and Use Committee of Northeast Electric Power University (Approval Number: NEEPU-IACUC-2024-0106). Laboratory Animal License Number: SYXK (Hubei) 2023-0101. This laboratory has been certified under the GB/T 19001-2016/ISO 9001:2015 Quality Management System (Certificate No. 0350622030307R1M).
